# Creeping in the night: What might ecologists be missing?

**DOI:** 10.1371/journal.pone.0198277

**Published:** 2018-06-13

**Authors:** Carol Anne Nichols, Kathleen Alexander

**Affiliations:** 1 Department of Fish and Wildlife Conservation, Virginia Tech, Blacksburg, VA, United States of America; 2 Centre for Conservation of African Resources, Animals, Communities, and Land use (CARACAL), Kasane, Botswana; University of Southern California, UNITED STATES

## Abstract

Wildlife activity patterns tend to be defined by terms such as diurnal and nocturnal that might not fully depict the complexity of a species’ life history strategy and behavior in a given system. These activity pattern categories often influence the methodological approaches employed, including the temporal period of study (daylight or nighttime). We evaluated banded mongoose (*Mungos mungo*) behavior in Northern Botswana through the use of remote sensing cameras at active den sites in order to characterize early morning behavior for this diurnal species. Our approach, however, provided the facility to capture unexpected nocturnal activity in a species that had otherwise only been studied during daylight hours. Camera traps were deployed for 215 trap days (24 hour data capture period) at den sites, capturing 5,472 photos over all events. Nocturnal activity was identified in 3% of trap days at study den sites with both vigilant and non-vigilant nocturnal behaviors identified. While vigilant behaviors involved troop fleeing responses, observations of non-vigilant behaviors suggest nonresident mongoose may investigate den sites of other troops during nocturnal time periods. There was no association between the occurrence of nocturnal activity and lunar phase (Fisher’s exact test, n = 215, p = 0.638) and thus, increased moonlight was not identified as a factor influencing nocturnal behavior. The drivers and fitness consequences of these nocturnal activities remain uncertain and present intriguing areas for future research. Our findings highlight the need for ecological studies to more explicitly address and evaluate the potential for temporal variability in activity periods. Modifying our approach and embracing variation in wildlife activity patterns might provide new insights into the interaction between ecological phenomenon and species biology that spans the diurnal–nocturnal spectrum.

## Introduction

The categorization of wildlife activity tends to be constrained to traditional day-night niches, strictly classifying species as diurnal or nocturnal [1–3]. Less frequently, the term crepuscular is used, since many diurnal species are active at dawn and dusk as well as in full daylight [4]. Identification of distinct adaptations to either a diurnal or nocturnal life history strategy (e.g. larger orbits in nocturnal primate species improving night vision [5]) may contribute to the tendency by some to view species’ activity patterns as more of a binary trait, rather than extending across a spectrum of temporal activity types [2, 6]. The characterization of a species to an activity pattern may translate into a methodological approach where evaluation extending beyond the dominant activity period is never attempted.

This more simplistic approach, however, was questioned in the 1970s with the characterization of the activity patterns of the presumed diurnal Mayotte lemur (*Lemur fluvus mayottensis*). This species was observed to apportion its behavior equally between day and night periods [7]. As a consequence, the term cathemerality was developed to capture this temporal activity type, adding to the previously established, and mutually exclusive categories of nocturnal and diurnal. As a behavioral strategy, cathemerality is thought to allow considerable advantages, identifying the ability for the species to engage in diurnal and/or nocturnal activity periods in response to varying ecological conditions [3]. Factors such as temperature, access to food resources, and predation risk are all thought to be important in promoting cathemerality [3]. At least eight species of Madagascar lemur exhibit cathemeral activity patterns [1, 8, 9]. This new term was initially met with skepticism and even rejected by a reviewer in the peer review process as it was considered to be “unnecessary new jargon” [2]. This initial rejection illustrates the commitment to the notion that animal activity cycles can be viewed as if they are a binary trait, rather than extending across a spectrum of temporal activity types. Where behavioral activity is identified outside of the expected time period, these events are often considered unusual and, therefore, of potentially limited ecological significance. However, these events may provide information critical to understanding a species diel behavior and ecology in a given landscape.

A prominent hypothesis for diurnal species expanding activity into the night is that nighttime activity occurs due to elevated light levels [10–12]. Singing at night was typical of unmated northern mockingbirds (*Mimus polyglottos*) and regularly occurred during full moon periods and less frequently during other lunar phases [13]. For carnivores, more recent studies reveal that nocturnal activity may be more pronounced than previously thought (14,15). For traditionally perceived diurnal species such as the African wild dog (*Lycaon pictus* [14, 15]) and cheetahs (*Acinonyx jubatus* [15]), nocturnal activity for both species comprised roughly 25% of their overall activity budget [15]. Nocturnal activity was associated with the lunar cycle and restricted to brighter nights, potentially allowing these species to avoid stronger competitors such as lions (*Panthera leo*) and hyenas (*Crocuta crocuta*), which are active at night [15].

We used camera traps to study behavior among banded mongooses (*Mungos mungo*), a diurnal mesocarnivore [16], at den sites in Northern Botswana. Systematic studies of banded mongoose ecology and behavior previously have only been undertaken during daylight hours (e.g. [16–22]). Here, we report nocturnal activity for this presumed exclusively diurnal species. We characterize behavior type and test whether observed nocturnal activity patterns are associated with brighter lunar phases as observed in other species. We discuss our findings in the light of our current approach to activity pattern classification and methodological approaches for ecological and behavioral studies of wildlife.

## Methods

### Field methods

Remote sensing camera traps were deployed from January 4 to November 9, 2016 in order to study banded mongoose behavior at the den sites of 17 troops located in our long-term study site in Northern Botswana (S1 Table). The study area covered the urban areas of Kasane and Kazungula (defined as town e.g., tourist lodges, residential areas, camp kitchens, farm land, and garbage sites) as well as natural habitats (defined as park e.g., forest reserves and Chobe National Park). Animals were tracked through the use of radio collars as previously described [23]. Each radio collared troop (n = 15) was tracked five days a week, alternating eight one day and seven the next. Once the den site was located, camera traps were mounted on nearby fence posts, trees, poles, and occasionally man-made stake mounts to obtain the optimal position for photographing movement and behavior at the den site. While tracking the marked troops, we also opportunistically placed cameras at den sites (n = 2) that were identified, but the troops had not been fitted with a radio collar. A trap day was defined as a 24-hour monitoring period of banded mongoose activity at the den site.

The study was conducted under approval and in accordance with the guidelines of the Virginia Tech Institutional Care and Use Committee (16-217-FIW) and under permit of the Botswana Government.

### Photo classifications and moon phase

The trap events were categorized as occurring in the day or night according to the time stamp on the photographs. The hours used to classify trap events were based on astronomical twilight. Considering Botswana’s tropical location, sunrise and sunset do not drastically change throughout the year. The days are slightly shorter in the winter months from May to August. During the winter, the day was defined as occurring between the hours of 0500–2000, with night occurring between 2000 and 0500 hours. During the summer, the day capture period was lengthened to the hours between 0400 and 2100, and the night capture period was adjusted accordingly. A trap event that contained photographs during the nighttime hours was identified as a night trap event and further categorized by lunar phase (i.e. full, waning, waxing, and new). Waxing, waning, and full moon phases were grouped as moonlight nights. The new moon phases were considered dark nights.

### Data analysis

The proportion of nighttime activity was calculated as the number of trap days where nighttime activity was identified (night trap event) over the total number of trap days. Fisher’s exact test was used in RStudio (Version 0.99.484) to evaluate associations between night trap events and variables of interest. Given the limited number of night trap events observed, more complex approaches could not be employed and we, therefore, assume independence of observations.

## Results

From January to November 2016, nighttime activity was identified for 3% of the trap days (SD = 18%, n = 7/215) and across five of the 11 months of the study. Night trap events occurred between 2000 and 2400 hours (n = 7). The nighttime activity was observed in 29% of study troops (n = 5/17 study troops, S1 Table). There was no significance between land classification of the den site (town or park) and the occurrence of night activity (Fisher’s exact test p = 1). However, the limited number of park troops might have influenced our ability to detect an effect.

Banded mongoose nighttime activity was seen across all moon phases with no significant difference between the nights with no light (i.e., the new moon) and the phases (i.e. waning, waxing, full) that did provide moonlight (Fisher’s exact test p = 0.638; S1 Dataset for trap days and associated moon phases).

## Discussion

Like other social mongooses, banded mongooses are considered diurnal species [24] and, across studies, ecological and behavioral investigations have been restricted to daylight periods [16–22]. However, this limited temporal approach may fail to capture data critical to understanding the ecology, biology of a species, and the temporal nature of space use. Here, we report nocturnal behavior and space use for banded mongooses. Our observed nocturnal behaviors were both vigilant and non-vigilant in nature (S2 Table for ethogram). Where vigilance behaviors were identified, they were followed by fleeing and den relocation, which is unique for nighttime study periods in banded mongoose. Photos from the cameras traps did not, however, provide information as to what prompted these behaviors. Fleeing behavior in this species had only been previously been reported in association with intergroup aggression events [25].

Non-vigilant behaviors were also observed and included individuals walking towards an active den site, investigating a den site, and in one situation, an individual emerges from the den to chase a mongoose. In that last scenario, only one mongoose returns to the den hours later. In each of these events, photographic evidence of mongooses emerging from the den is absent (with exception of the mongoose chasing a putative intruder). Indeed in two of the instances, there was only one entrance to the den and photographic evidence of den emergence would have been necessary if the mongoose was from the resident troop. These data suggest that non-resident mongoose may make forays at night into the home range of other troops. Further research is needed to fully understand the ecological purpose of these behaviors.

When evaluating the lunar phases and nighttime activity, night trap events were identified across both the moonlight and dark nights. Nocturnal behavior in banded mongoose did not appear to be influenced by availability of light. Lack of association with moon phase suggests other important ecological factors or stimuli are encountered at night and influence the occurrence of these behaviors.

Fitness advantages for nocturnal behavior in diurnal species have been previously identified [26, 27], and signal the importance of identifying diel behavior patterns rather than focusing on the dominant activity period. For example, the diurnal yellow breasted chat (*Icterina virens*) will move outside of their territories at night with fertile females engaging in extrapair copulations, potentially allowing them to go undetected by their social mate [26]. While being primarily diurnal, brown bears (*Ursus arctos*) forage equally between daylight and darkness during salmon migrations, with capture success increasing during the night [27]. Restricting data collection for diurnal species to expected daytime activity periods might hinder or limit our ability to gain further insight into important aspects of the biology and ecology of wildlife species.

Focusing effort toward dominant activity periods may also result in erroneous conclusions when restricting data collection to a particular activity time. For example, non-invasive fecal glucocorticoids are often used to evaluate stress responses in wildlife [28] and results are often interpreted in conjunction with behavioral data collected during the same diurnal time period of collection or the diurnal period the day before collection [29]. Here, stress levels are interpreted and aligned with observed daytime movements and behaviors when, in fact, the stress response may reflect the occurrence of unobserved nocturnal events. Failure to account for variation in activity periods might not only undermine study objectives, but also the veracity of our understanding of a species’ ecology and behavior.

Considering that heterogeneity is a fundamental underlying phenomena of ecology [30], temporal variation in activity patterns should be explicitly accounted for in methodological approaches. Our observations of banded mongooses illustrate the importance of including observations during low activity times when studying a wildlife species. Initially, ecology assumed spatial homogeneity for convenience and simplicity, with heterogeneity an unwelcome but necessary complication [31]. Study approaches and theoretical frameworks in the field of landscape ecology that have embraced heterogeneity and complexity have provided new and critical insights into key ecological processes (e.g. community dynamics or succession, the role of edges, patch dynamics)[31]. A similar inclusion of heterogeneity into our consideration of activity periods and associated behaviors is called for and will likely lead to a new era of ecological discovery.

## Supporting information

S1 Table The occurrence of nocturnal behavior across banded mongoose troops in Northern Botswana. Study troops are listed by land class and month of nocturnal behavior, if this was observed.
(DOCX)

S2 Table Ethogram of banded mongoose behavior. Mongoose behaviors were identified and categorized as being either non-vigilant or vigilant.
(DOCX)

S1 Dataset Banded mongoose photo trap data and activity period. A total of 215 trap days were recorded across seventeen troops. The occurrence of nocturnal activity and moon phase (i.e. full, waning, waxing, and new) is identified.
(DOCX)
