# Supplementary material for: Creeping in the night: What might ecologists be missing?
Source: PLoS One. 2018 Jun 13;13(6):e0198277. doi: 10.1371/journal.pone.0198277 (PMC5999080; doi:10.1371/journal.pone.0198277)
Supplement: S1 Table — Study troops are listed by land class and month of nocturnal behavior, if this was observed. (DOCX) [file pone.0198277.s001.docx]

| Troop | General Land Class | Nighttime Activity | Month of Nighttime Activity |
| --- | --- | --- | --- |
| Chobe Chilwero | Park | No | NA |
| Chobe Game Lodge | Park | No | NA |
| Chobe Safari Lodge | Town | Yes | January  July |
| Dumpsite/Chobe Farms | Town | No | July |
| Forestry Department | Town | Yes | January  June |
| Kwalape Lodge | Town | No | NA |
| Kubu Lodge | Town | No | NA |
| Mowana Break-off | Town | No | NA |
| Mowana Lodge | Town | No | NA |
| Park Gates | Park | No | NA |
| Plateau | Town | No | NA |
| Sedudu Riverfront | Park | No | NA |
| Sedudu Valley | Park | Yes | March |
| Thebe Lodge/ Sefelana | Town | No | NA |
| Water Affairs | Town | No | NA |
| Watercart East | Park | No | NA |
| Watercart West | Park | Yes | October |
