# Supplementary material for: Creeping in the night: What might ecologists be missing?
Source: PLoS One. 2018 Jun 13;13(6):e0198277. doi: 10.1371/journal.pone.0198277 (PMC5999080; doi:10.1371/journal.pone.0198277)
Supplement: S2 Table — Mongoose behaviors were identified and categorized as being either non-vigilant or vigilant. (DOCX) [file pone.0198277.s002.docx]

| Category | Behavior | Description |
| --- | --- | --- |
| Non-vigilant | Forage | Digging for food item, manipulating food item, chewing |
|  | Running | Trotting or running |
|  | Walking | Walking |
|  | Play (alone) | Manipulating non-food object, usually interspersed with bouncing and play calls |
|  | Contact garbage or human food | Contact with garbage or human food, primarily while foraging in it |
|  | Emerge | Emerging from den or other structure |
|  | Excavate | Digging at den site. Differs from digging for food in that both front paws are usually used synchronously for excavating, the depth is usually greater than for foraging, and no food items are eaten. |
|  | Social play | Rough and tumble contact or chasing interspersed with bouncing and play call |
|  | Aggression/defense | Agonistic contact with at least one other mongoose. Distinguished from play by aggressive sounds, lack of bouncing, and more forceful motions that may cause injury (e.g. forceful scratching and biting rather than pawing and mouthing) |
|  | Copulate | Mating |
|  | Allofeed | Feeding another mongoose, typically a pup |
|  | Jerky greeting | A greeting where two animals jerk their heads back and forth while approaching each other, culminating in bodily contact with continued jerking of the head |
|  | Clasp | One mongoose clasping another with front paws, similar to a mating position, but without intromission. Often performed during play. |
|  | Beg | Performed by pups and sub adults. Following and sometimes touching another mongoose while emitting begging calls. |
|  | Lead | An individual incites the group to move from the current location with contact calls that are louder and faster than usual while walking or trotting away from the current location. |
|  | Follow | Walking or trotting in a direction that the leading animal is moving. Usually accompanied by mimicking the leading call. |
|  | Lying down | Lying down with very little movement, whether asleep or awake. |
|  | Sit or stand still | Sitting or standing with little to no movement. Head is below horizontal |
|  | Groom | Scratching, gnawing, or licking oneself. (Counted as resting behavior because generally interspersed with lying down or sitting during periods when the troop is resting) |
|  | Allogroom | Licking or gnawing another mongoose. Typically performed during a troop resting periods. |
|  | Social rest | Lying while in physical contact with at least one other mongoose |
|  | Social sit or stand | Sitting or standing while in physical contact at least one other mongoose |
|  | Smell | Smelling the ground or an object |
|  | Scent smell | Smelling an area that is known to be scent marked |
|  | Social smell | Smelling another mongoose |
|  | Defecate/urinate | Excretion |
|  | Token dance urination | Urination involving the stereotypical stomping of ground with hind limbs |
|  | Latrine Defecation | Feces are deposited in token amounts at specific sites called latrines |
|  | Social scent mark | Rubbing another mongoose with anal glands |
|  | Scent mark | Rubbing ground or object with anal glands |
|  | Over-marking | One individual places its scent mark directly over the scent mark of another individual |
|  | Cheek mark | Rubbing the side of the ‘face’ on surfaces and along the ground |
| Vigilance Behaviors | Vigilance | Head is horizontal or above horizontal but not oriented to another mongoose.  Head bobbing may occur. Gaze may be direct toward a perceived threat. All four legs are on the ground. |
|  | Bipedal Vigilance | Head is horizontal or above horizontal but not oriented to another mongoose  Head bobbing may occur. Gaze may be direct toward a perceived threat. Standing on back two legs. |
|  | Scanning | Head is horizontal or above horizontal and scans the landscape. |
|  | Flee | Trotting or running from a perceived threat |
|  | Alarm call | Any of several calls used when a perceived threat is present. Often accompanied by vigilance or fleeing. |
|  | Vigilance while chewing | Exhibits vigilance behaviors while chewing |
