# Supplementary material for: Creeping in the night: What might ecologists be missing?
Source: PLoS One. 2018 Jun 13;13(6):e0198277. doi: 10.1371/journal.pone.0198277 (PMC5999080; doi:10.1371/journal.pone.0198277)
Supplement: S1 Dataset — A total of 215 trap days were recorded across seventeen troops. The occurrence of nocturnal activity and moon phase (i.e. full, waning, waxing, and new) is identified. (DOCX) [file pone.0198277.s003.docx]

| Trap Day | Troop(s) Sampled | Night activity recorded | Moon phase | Time |
| --- | --- | --- | --- | --- |
| 3-Jan-16 | Water Affairs | - | Waning | - |
| 4-Jan-16 | Water Affairs | - | Waning | - |
| 5-Jan-16 | Water Affairs | - | Waning | - |
| 6-Jan-16 | Water Affairs | - | New | - |
| 7-Jan-16 | Forestry, Mowana, Water Affairs | - | New | - |
| 10-Jan-16 | Water Affairs | - | New | - |
| 14-Jan-16 | Watercart East | - | Waxing | - |
| 16-Jan-16 | Chobe Safari Lodge | - | Waxing | - |
| 18-Jan-16 | Chobe Safari Lodge | - | Waxing | - |
| 19-Jan-16 | Chobe Safari Lodge | - | Waxing | - |
| 20-Jan-16 | Chobe Safari Lodge | Night | Waxing | 2342 |
| 21-Jan-16 | Chobe Safari Lodge, Forestry | - | Full | - |
| 22-Jan-16 | Chobe Safari Lodge, Forestry | - | Full | - |
| 23-Jan-16 | Chobe Safari Lodge, Forestry | Night | Full | 2100 |
| 24-Jan-16 | Forestry | - | Full | - |
| 25-Jan-16 | Forestry, Kubu, Mowana break-off | - | Full | - |
| 26-Jan-16 | Forestry, Kubu, Mowana break-off | - | Full | - |
| 27-Jan-16 | Mowana break-off | - | Full | - |
| 28-Jan-16 | Forestry, Mowana break-off | - | Waning | - |
| 29-Jan-16 | Chobe Chilwero, Forestry, Mowana break-off | - | Waning | - |
| 30-Jan-16 | Forestry | - | Waning | - |
| 5-Feb-16 | Mowana | - | New | - |
| 6-Feb-16 | Mowana | - | New | - |
| 7-Feb-16 | Mowana | - | New | - |
| 9-Feb-16 | Chobe Safari Lodge | - | New | - |
| 10-Feb-16 | Chobe Safari Lodge | - | New | - |
| 11-Feb-16 | Forestry | - | New | - |
| 18-Feb-16 | Chobe Safari Lodge, Water Affairs | - | Waxing | - |
| 19-Feb-16 | Chobe Safari Lodge | - | Full | - |
| 22-Feb-16 | Mowana | - | Full | - |
| 23-Feb-16 | Mowana, Sedudu Riverfront | - | Full | - |
| 24-Feb-16 | Sedudu Riverfront | - | Full | - |
| 25-Feb-16 | Sedudu Riverfront | - | Full | - |
| 3-Mar-16 | Watercart East | - | Waning | - |
| 4-Mar-16 | Sedudu Valley | - | Waning | - |
| 5-Mar-16 | Sedudu Valley | - | New | - |
| 6-Mar-16 | Sedudu Valley | Night | New | 2238 |
| 7-Mar-16 | Sedudu Valley | - | New | - |
| 12-Mar-16 | Sefelana | - | Waxing | - |
| 14-Mar-16 | Mowana | - | Waxing | - |
| 15-Mar-16 | Sefelana | - | Waxing | - |
| 16-Mar-16 | Sefelana | - | Waxing | - |
| 17-Mar-16 | Mowana, Sefelana | - | Waxing | - |
| 19-Mar-16 | Chobe Chilwero, Sefelana | - | Waxing | - |
| 20-Mar-16 | Sefelana | - | Full | - |
| 21-Mar-16 | Chobe Chilwero, Mowana, Sefelana | - | Full | - |
| 22-Mar-16 | Mowana, Sefelana | - | Full | - |
| 23-Mar-16 | Chobe Chilwero | - | Full | - |
| 24-Mar-16 | Chobe Chilwero | - | Full | - |
| 25-Mar-16 | Chobe Chilwero | - | Full | - |
| 4-Apr-16 | Chobe Safari Lodge, Forestry | - | New | - |
| 5-Apr-16 | Forestry, Water Affairs | - | New | - |
| 6-Apr-16 | Forestry, Water Affairs | - | New | - |
| 7-Apr-16 | Forestry, Sefelana, Water Affairs | - | New | - |
| 8-Apr-16 | Kubu, Sefelana, Water Affairs | - | New | - |
| 9-Apr-16 | Water Affairs | - | New | - |
| 10-Apr-16 | Water Affairs | - | New | - |
| 11-Apr-16 | Chobe Game Lodge, Water Affairs | - | Waxing | - |
| 12-Apr-16 | Water Affairs | - | Waxing | - |
| 13-Apr-16 | Chobe Game Lodge, Water Affairs | - | Waxing | - |
| 14-Apr-16 | Kubu, Water Affairs | - | Waxing | - |
| 15-Apr-16 | Chobe Game Lodge, Water Affairs | - | Waxing | - |
| 16-Apr-16 | Chobe Game Lodge, Water Affairs | - | Waxing | - |
| 18-Apr-16 | Chobe Game Lodge | - | Full | - |
| 19-Apr-16 | Chobe Game Lodge, Kubu | - | Full | - |
| 20-Apr-16 | Chobe Game Lodge | - | Full | - |
| 21-Apr-16 | Chobe Game Lodge, Mowana | - | Full | - |
| 22-Apr-16 | Chobe Game Lodge, Kubu | - | Full | - |
| 23-Apr-16 | Chobe Game Lodge, Kubu | - | Full | - |
| 24-Apr-16 | Chobe Game Lodge | - | Full | - |
| 25-Apr-16 | Chobe Game Lodge | - | Full | - |
| 26-Apr-16 | Sedudu Riverfront | - | Waning | - |
| 27-Apr-16 | Chobe Safari Lodge, Forestry, Mowana Break-off | - | Waning | - |
| 28-Apr-16 | Chobe Game Lodge | - | Waning | - |
| 29-Apr-16 | Dumpsite/Chobe Farms, Mowana | - | Waning | - |
| 1-May-16 | Mowana | - | Waning | - |
| 2-May-16 | Chobe Game Lodge, Dumpsite/Chobe Farms | - | Waning | - |
| 3-May-16 | Mowana | - | New | - |
| 4-May-16 | Mowana | - | New | - |
| 5-May-16 | Chobe Chilwero | - | New | - |
| 6-May-16 | Mowana | - | New | - |
| 11-May-16 | Dumpsite/Chobe Farms | - | Waxing | - |
| 12-May-16 | Water Affairs | - | Waxing | - |
| 23-May-16 | Water Affairs | - | Full | - |
| 28-May-16 | Kubu | - | Waning | - |
| 29-May-16 | Chobe Safari Lodge | - | Waning | - |
| 30-May-16 | Chobe Safari Lodge, Mowana, Mowana Break-off | - | Waning | - |
| 31-May-16 | Chobe Safari Lodge, Mowana Break-off | - | Waning | - |
| 1-Jun-16 | Chobe Safari Lodge, Sefelana | - | Waning | - |
| 2-Jun-16 | Chobe Safari Lodge | - | New | - |
| 3-Jun-16 | Chobe Safari Lodge, Water Affairs | - | New | - |
| 4-Jun-16 | Mowana Break-off | - | New | - |
| 6-Jun-16 | Kubu, Mowana | - | New | - |
| 8-Jun-16 | Chobe Game Lodge, Chobe Safari Lodge | - | New | - |
| 9-Jun-16 | Chobe Game Lodge | - | Waxing | - |
| 10-Jun-16 | Kubu | - | Waxing | - |
| 11-Jun-16 | Chobe Safari Lodge | - | Waxing | - |
| 12-Jun-16 | Kubu | - | Waxing | - |
| 13-Jun-16 | Chobe Game Lodge, Chobe Safari Lodge, Forestry, Water Affairs, Mowana Break-off | - | Waxing | - |
| 14-Jun-16 | Chobe Game Lodge, forestry, Water Affairs, Mowana Break-off | - | Waxing | - |
| 15-Jun-16 | Chobe Game Lodge, Forestry | - | Waxing | - |
| 16-Jun-16 | Chobe Game Lodge, Forestry, Sedudu Riverfront | - | Full | - |
| 17-Jun-16 | Chobe Chilwero, Chobe Game Lodge, Sedudu Riverfront | - | Full | - |
| 18-Jun-16 | Chobe Chilwero, Chobe Game Lodge, Sedudu Riverfront | - | Full | - |
| 19-Jun-16 | Chobe Safari Lodge, Forestry | - | Full | - |
| 20-Jun-16 | Chobe Chilwero, Chobe Game Lodge, Kubu, Sedudu Riverfront | - | Full | - |
| 22-Jun-16 | Chobe Chilwero, Chobe Game Lodge, Plateau | - | Full | - |
| 23-Jun-16 | Chobe Game Lodge, Plateau, Sedudu Riverfront | - | Full | - |
| 24-Jun-16 | Chobe Game Lodge, Park Gates, Plateau, Sedudu Riverfront | - | Waning | - |
| 25-Jun-16 | Chobe Game Lodge, Forestry, Plateau, Sedudu Riverfront, Sefelana | - | Waning | - |
| 26-Jun-16 | Chobe Game Lodge, Forestry | - | Waning | - |
| 27-Jun-16 | Chobe Chilwero, Chobe Game Lodge, Forestry | - | Waning | - |
| 28-Jun-16 | Chobe Chilwero, Chobe Game Lodge, Dumpsite/Chobe Farms, Forestry, Sedudu Riverfront | Night | Waning | 2058; 2245 |
| 29-Jun-16 | Chobe Game Lodge, Dumpsite/Chobe Farms, Forestry, Sefelana | - | Waning | - |
| 30-Jun-16 | Chobe Chilwero, Chobe Safari Lodge, Mowana, Sedudu Riverfront, Water Affairs | - | Waning | - |
| 1-Jul-16 | Chobe Chilwero, Chobe Game Lodge, Plateau, Sedudu Riverfront, Water Affairs | - | New | - |
| 2-Jul-16 | Dumpsite/Chobe Farms, Chobe Game Lodge, Sedudu Riverfront, Water Affairs | - | New | - |
| 3-Jul-16 | Dumpsite/Chobe Farms, Chobe Game Lodge, Mowana, Plateau, Sedudu Riverfront | - | New | - |
| 4-Jul-16 | Chobe Chilwero, Dumpsite/Chobe Farms, Plateau, Sedudu Riverfront | - | New | - |
| 5-Jul-16 | Forestry, Kubu, Sedudu Riverfront | - | New | - |
| 6-Jul-16 | Chobe Chilwero, Kwalape, Mowana | - | New | - |
| 7-Jul-16 | Dumpsite/Chobe Farms, Kwalape, Mowana | - | New | - |
| 8-Jul-16 | Chobe Chilwero, Dumpsite/Chobe Farms, Chobe Safari Lodge, Mowana, Plateau | - | New | - |
| 9-Jul-16 | Plateau | - | Waxing | - |
| 10-Jul-16 | Chobe Chilwero, Plateau | - | Waxing | - |
| 11-Jul-16 | Chobe Chilwero, Mowana, Plateau | - | Waxing | - |
| 12-Jul-16 | Mowana | - | Waxing | - |
| 13-Jul-16 | Chobe Chilwero, Dumpsite/Chobe Farms, Forestry, Kwalape | - | Waxing | - |
| 14-Jul-16 | Forestry, Kwalape | - | Waxing | - |
| 15-Jul-16 | Chobe Chilwero, Dumpsite/Chobe Farms, Forestry, Kwalape | - | Waxing | - |
| 16-Jul-16 | Chobe Chilwero, Chobe Safari Lodge, Forestry, Kwalape | - | Full | - |
| 17-Jul-16 | Chobe Chilwero, Dumpsite/Chobe Farms, Chobe Safari Lodge, Forestry, Plateau | - | Full | - |
| 18-Jul-16 | Chobe Chilwero, Chobe Safari Lodge, Plateau | - | Full | - |
| 19-Jul-16 | Chobe Chilwero, Dumpsite/Chobe Farms, Chobe Safari Lodge, Forestry, Plateau | - | Full | - |
| 20-Jul-16 | Chobe Chilwero, Forestry, Kwalape, Plateau | - | Full | - |
| 21-Jul-16 | Dump, Kwalape, Plateau | - | Full | - |
| 22-Jul-16 | Chobe Chilwero, Forestry, Kubu, Kwalape, Plateau, Sedudu Riverfront | - | Full | - |
| 23-Jul-16 | Chobe Chilwero, Forestry, Kwalape, Plateau | - | Waning | - |
| 24-Jul-16 | Dumpsite/Chobe Farms, Forestry, Kwalape, Plateau | - | Waning | - |
| 25-Jul-16 | Dumpsite/Chobe Farms, Kwalape, Sedudu Riverfront | Night | Waning | 2054 |
| 26-Jul-16 | Dumpsite/Chobe Farms, Forestry, Kwalape, Plateau, Sedudu Riverfront | - | Waning | - |
| 28-Jul-16 | Chobe Chilwero, Dumpsite/Chobe Farms, Chobe Safari Lodge, Forestry, Plateau | Night | Waning | 2059; 2315 |
| 29-Jul-16 | Chobe Chilwero, Chobe Safari Lodge, Forestry, Sedudu Riverfront | - | Waning | - |
| 30-Jul-16 | Chobe Chilwero, Dumpsite/Chobe Farms, Forestry | - | Waning | - |
| 31-Jul-16 | Dumpsite, Sedudu Riverfront | - | New | - |
| 1-Aug-16 | Chobe Chilwero, Forestry, Sedudu Riverfront | - | New | - |
| 2-Aug-16 | Chobe Chilwero, Dumpsite/Chobe Farms, Forestry, Sedudu Riverfront | - | New | - |
| 3-Aug-16 | Chobe Chilwero, Dumpsite/Chobe Farms, Sedudu Riverfront | - | New | - |
| 4-Aug-16 | Chobe Chilwero, Dumpsite/Chobe Farms, Forestry, Kubu, Sedudu Riverfront | - | New | - |
| 5-Aug-16 | Chobe Chilwero, Chobe Safari Lodge, Forestry, Kubu | - | New | - |
| 6-Aug-16 | Chobe Chilwero, Chobe Safari Lodge, Sedudu Riverfront | - | New | - |
| 7-Aug-16 | Chobe Chilwero, Forestry, Sedudu Riverfront | - | Waxing | - |
| 8-Aug-16 | Chobe Chilwero, Chobe Safari Lodge, Kwalape, Sedudu Riverfront | - | Waxing | - |
| 9-Aug-16 | Forestry, Sedudu Riverfront | - | Waxing | - |
| 10-Aug-16 | Chobe Chilwero, Forestry, Sedudu Riverfront | - | Waxing | - |
| 11-Aug-16 | Forestry, Sedudu Riverfront | - | Waxing | - |
| 12-Aug-16 | Chobe Chilwero, Forestry, Sedudu Riverfront | - | Waxing | - |
| 13-Aug-16 | Sedudu Riverfront | - | Waxing | - |
| 18-Aug-16 | Kubu | - | Full | - |
| 23-Aug-16 | Chobe Chilwero | - | Waning | - |
| 24-Aug-16 | Dumpsite/Chobe Farms | - | Waning | - |
| 25-Aug-16 | Sedudu Riverfront | - | Waning | - |
| 26-Aug-16 | Chobe Safari Lodge | - | Waning | - |
| 27-Aug-16 | Chobe Safari Lodge, Sedudu Riverfront | - | Waning | - |
| 28-Aug-16 | Chobe Safari Lodge | - | Waning | - |
| 29-Aug-16 | Mowana, Sedudu Riverfront | - | New | - |
| 30-Aug-16 | Mowana, Sedudu Riverfront | - | New | - |
| 31-Aug-16 | Chobe Safari Lodge, Sedudu Riverfront | - | New | - |
| 1-Sep-16 | Chobe Safari Lodge, Water Affairs | - | New | - |
| 2-Sep-16 | Kwalape, Sedudu Riverfront | - | New | - |
| 3-Sep-16 | Chobe Safari Lodge | - | New | - |
| 6-Sep-16 | Water Affairs | - | Waxing | - |
| 7-Sep-16 | Chobe Chilwero | - | Waxing | - |
| 8-Sep-16 | Chobe Chilwero | - | Waxing | - |
| 9-Sep-16 | Sefelana | - | Waxing | - |
| 12-Sep-16 | Sefelana | - | Waxing | - |
| 13-Sep-16 | Sefelana | - | Full | - |
| 14-Sep-16 | Kubu | - | Full | - |
| 23-Sep-16 | Watercart East | - | Waning | - |
| 24-Sep-16 | Chobe Chilwero | - | Waning | - |
| 25-Sep-16 | Watercart East | - | Waning | - |
| 26-Sep-16 | Chobe Chilwero, Watercart East | - | Waning | - |
| 27-Sep-16 | Chobe Chilwero | - | Waning | - |
| 28-Sep-16 | Chobe Chilwero | - | New | - |
| 29-Sep-16 | Chobe Chilwero, Kwalape, Watercart East | - | New | - |
| 30-Sep-16 | Watercart East | - | New | - |
| 1-Oct-16 | Watercart East | - | New | - |
| 10-Oct-16 | Water Affairs, Mowana | - | Waxing | - |
| 12-Oct-16 | Water Affairs | - | Waxing | - |
| 14-Oct-16 | Chobe Chilwero | - | Full | - |
| 15-Oct-16 | Chobe Chilwero | - | Full | - |
| 16-Oct-16 | Chobe Chilwero | - | Full | - |
| 17-Oct-16 | Chobe Chilwero | - | Full | - |
| 18-Oct-16 | Watercart East | - | Full | - |
| 19-Oct-16 | Watercart East | - | Full | - |
| 20-Oct-16 | Watercart East | - | Waning | - |
| 21-Oct-16 | Water Affairs, Watercart East | - | Waning | - |
| 22-Oct-16 | Chobe Chilwero, Water Affairs, Watercart East | - | Waning | - |
| 23-Oct-16 | Chobe Chilwero, Water Affairs, Watercart East | - | Waning | - |
| 24-Oct-16 | Water Affairs | - | Waning | - |
| 25-Oct-16 | Water Affairs, Watercart West | Night | Waning | 2256 |
| 26-Oct-16 | Water Affairs | - | Waning | - |
| 27-Oct-16 | Chobe Game Lodge, Water Affairs | - | New | - |
| 28-Oct-16 | Dumpsite/Chobe Farms, Kwalape, Water Affairs | - | New | - |
| 29-Oct-16 | Dumpsite/Chobe Farms, Kwalape, Water Affairs | - | New | - |
| 30-Oct-16 | Dumpsite/Chobe Farms, Kwalape, Water Affairs | - | New | - |
| 31-Oct-16 | Dumpsite/Chobe Farms, Kwalape, Water Affairs | - | New | - |
| 1-Nov-16 | Water Affairs | - | New | - |
| 2-Nov-16 | Dumpsite/Chobe Farms | - | New | - |
| 3-Nov-16 | Dumpsite/Chobe Farms | - | New | - |
| 4-Nov-16 | Dumpsite/Chobe Farms, Kubu, Mowana Break-off, Plateau | - | Waxing | - |
| 5-Nov-16 | Mowana Break-off, Plateau | - | Waxing | - |
| 6-Nov-16 | Dumpsite/Chobe Farms, Mowana Break-off, Plateau | - | Waxing | - |
| 7-Nov-16 | Dumpsite/Chobe Farms, Mowana Break-off | - | Waxing | - |
| 9-Nov-16 | Dumpsite/Chobe Farms | - | Waxing | - |
